# Modelling sterile insect technique to control the population of *Anopheles gambiae*

**DOI:** 10.1186/s12936-015-0587-5

**Published:** 2015-02-22

**Authors:** James E Gentile, Samuel SC Rund, Gregory R Madey

**Affiliations:** University of Notre Dame, Cushing Hall, Notre Dame, USA; Centre for Immunity, Infection and Evolution, School of Biological Sciences, University of Edinburgh, Edinburgh, UK

**Keywords:** Agent-based modelling, SIT, Release of insects with a dominant lethal gene (RIDL)

## Abstract

**Background:**

There is a renewed effort to develop novel malaria control strategies as even well-implemented existing malaria control tools may fail to block transmission in some regions. Currently, transgenic implementations of the sterile insect technique (SIT) such as the release of insects with a dominant lethal, homing endonuclease genes, or *flightless* mosquitoes are in development. These implementations involve the release of transgenic male mosquitoes whose matings with wild females produce either no viable offspring or no female offspring. As these technologies are all in their infancy, little is known about the relative efficiencies of the various implementations.

**Methods:**

This paper describes agent-based modelling of emerging and theoretical implementations of transgenic SIT in *Anopheles gambiae* for the control of malaria. It reports on female suppression as it is affected by the SIT implementation, the number of released males, and competitiveness of released males.

**Conclusions:**

The simulation experiments suggest that a late-acting bisex lethal gene is the most efficient of the four implementations we simulated. They demonstrate 1) the relative impact of release size on a campaign’s effectiveness 2) late-acting genes are preferred because of their ability to exploit density dependent larval mortality 3) late-acting bisex lethal genes achieve elimination before their female-killing counterparts.

## Introduction

Mosquito-borne illnesses including dengue fever, lymphatic filariasis (elephantiasis), yellow fever, and malaria make up 16% of the global disease burden, particularly so in the developing world [1]. Of these, malaria accounts for 18% of childhood deaths in sub-Saharan Africa [2] and in 2010 afflicted 219 million people and resulted in 660,000 deaths [3]. Malaria has been successfully controlled in many regions through vector-targeted intervention such as insecticide-treated bed nets (ITNs) and indoor residual sprays (IRS). However, these interventions will fail to eliminate malaria in regions with extremely high rates of parasite transmission and in areas where mosquito vectors are not susceptible to existing control techniques (such as by exophily or insecticide resistance) [4].

Sterile Insect Technique (SIT) is one control strategy that is gaining renewed interest for the control of mosquito populations [5-7]. The technique involves the mass release of males sterilized through radiological or chemical means. These mate with the wild population by out-competing non-sterile wild males [8]. Females mosquitoes (generally) mate only once, thus a successful mating with a sterile male will prevent the development of any offspring from the inseminated female [9,10]. In some insect pests such as the tsetse fly [11], medfly [12], and melon fly [13] SIT has proved enormously successful in achieving local control or elimination, including eradication of the screwworm from all of North America [14]. In mosquitoes, over two dozen SIT trials have been reported; however, issues such as poor competition with wild males, semi-sterility, or no ultimate adult population reduction - even despite successful sterile matings have been reported, reviewed in Benedict and Robinson 2003 [15].

Promising new advances in mosquito population control using the release of transgenic, instead of chemically or radioactively sterilized mosquitoes, are now garnering substantial interest [6]. These transgenic implementations are extensions of SIT, in that released males mate with wild-type females to abnormal results due to the males carrying a cell-lethal transgene. These implementations may allow for straightforward mass-rearing of a male-only population for release; maintain larval competition with wild type mosquitoes; extend the “lifetime" of the intervention via propagation of the transgene through the population; and/or allow for the ability to induce or suppress the lethal trait through larval chemical exposure [16-19]. This paper broadly places these transgenic SIT implementations into one of four categories:

1. Early acting bisex (EBS) which is most similar to classical SIT whereby wild-type females mating with released males will produce no offspring. For modelling purposes, EBS is described as any implementation that involves the release of male mosquitoes modified such that no viable offspring (including larvae) are produced.
2. Early acting female-killing (EFK) whereby wild-type females mating with released males will produce no female offspring, but the transgene can be passed on through male progeny.
3. Late acting bisex (LBS) whereby wild-type females mating with released males will produce offspring that only survive through the aquatic stage and die shortly prior to or after emergence. Transgenic larvae that will eventually die prior to adulthood provide larval competition reducing wild-type larvae’s chances of survival.
4. Late acting female-killing (LFK) whereby wild-type females mating with released males will produce offspring, but only male offspring survive to adulthood where they may propagate the transgene to their progeny. Transgenic female larvae that will eventually die prior to adulthood provide larval competition to wild-type larvae.

Release of insects carrying a dominant lethal gene (RIDL) is a transgenic implementation that has received the most recent attention. Thomas *et al.* and Heinrich *et al.,* [20,21] reported early success in the development of RIDL, generating strains of *Drosophila* with cell-lethal gene products under chemically repressible promoters expressed either in females only, or with female specific toxicity. Since then, the generation of two late acting RIDL strains of the dengue fever mosquito, *Aedes aegypti*, has been reported. This includes an EBS implementation with a repressible strain that kills all larvae, leaving no viable offspring [22]. This strain has been shown to compete reasonably well with the wild-type, with only a 5% reduction in survivability, a 4-day shortened average lifespan, and (perhaps beneficially) a one-day earlier emergence as an adult [23]. The other RIDL strain is a LFK implementation where adult females die immediately due to their inability to fly, whereas males remain to propagate the transgene [24]. RIDL strains are currently in trials, with early success reported in both large-cage [25] and field trials [26-28].

The success of SIT implementations is dependent on wild-type and mass-reared mosquitoes readily mating [29], although experience from mass-rearing campaigns of agricultural pests such as the screwworm has shown that significant loss of mating competitiveness can arise in mass-reared populations [30]. Some processes to sterilize mosquitoes (e.g. irradiation) can induce a reduction in mating competitiveness [31] but transgenic techniques can generate a line of sterile mosquitoes with no loss in mating competitiveness. This has been shown in *Anopheles stephensi*, *Anopheles arabiensis*, and *Ae. aegypti* when compared against the parent (non-transgenic) lab colonies [32-34]. However, when lab-reared transgenic *Anopheles gambiae* mosquitoes were compared in large-cage field trials against wild collected mosquitoes, reductions in mating competitiveness were indeed noted, although competitiveness was still better than those achieved and accepted for use in the medfly control programs [35]. Taking into account the mating competitiveness of transgenic-mass reared mosquitoes is therefore another important consideration to make when planning or considering the implementation of an SIT campaign, especially when determining the mosquito release size which may need to be increased to counteract reduced competitiveness.

Much work on the malaria mosquito, *An. gambiae*, remains to be done. However, there are early and promising successes. Recent work developing mosquitoes carrying homing endonucleases (HEG) has shown progress [17,36]. Various implementations of homing endonucleases work by selectively destroying the X chromosome– preventing female offspring (an EFK implementation) or genes vital to males and/or females. Additionally, Thailayil *et al.*, [37], successfully demonstrated an EBS implementation using RNAi to knockdown the production of sperm.

This manuscript reports the first use of agent-based modelling to evaluate four implementations for the control of *An. gambiae* populations through the release of transgenic male mosquitoes at various release proportions and mating competitiveness rates. This work complements previous efforts to model SIT implementations which are summarized in Table 1. Agent-based modelling is used to simulate frequent releases of male transgenic mosquitoes homozygous for a cell-lethal transgene. The transgene exclusively kills the intended individuals 100% of the time (i.e. LFK will kill no males, kills all females, and no early larvae die). The results provide additional evidence that transgenic implementations of SIT could be used with success to eliminate *An. gambiae* vector populations, and estimate the relative success of various implementation strategies.

**Table 1:** **A survey of SIT modelling literature**

	**Biological observation**	**Model notes**
[38] Foster *et al.* 1988	Modelled EBS and female-killing of a	Computational model that works on
	hypothetical insect population at various	discrete generations comparing each male
	migrations, release rates, incomplete sterilities,	genotype with each female genotype.
	and number of mutated alleles. Under most,	
	but not all scenarios, EBS achieves better	
	control than female-killing.	
[39] Schliekelman and Gould 2000*a*	The authors model a hypothetical transgenic	The model uses combinatorics to determine
	implementation in hypothetical insects	a population’s genetic make-up as inherited
	whereby there are multiple lethal genes	from parents. Lethality is operational in a
	in released insects and these lethal genes	population subset with the correct allele
	are conditional, killing only when certain	active in their genotype.
	conditions are met and otherwise propagate. Found that under ideal conditions, this	
	implementation can be far more effective	
	than traditional EBS.	
[40] Schliekelman and Gould 2000*b*	Modelled transgenic implementation whereby	This model maintains 20 population signals,
	2–20 lethal genes were engineered into a	one for each possible active allele.
	hypothetical insect. As the number of lethal	Inheritance is captured as generations
	genes per released animal increases, there is a	inherit their genetic makeup from the
	greater chance any one progeny will inherit a	previous generation.
	lethal gene. Found under ideal conditions,	
	control could be achieved at rates several	
	orders of magnitude more effectively than	
	single gene EBS.	
[41] Barclay 2001	Modelled EBS in hypothetical insects, with	The analysis is performed with a discrete-
	special regard to incomplete sterility and lack	time population model. The paper reports
	of competitive mating ability, which cause	on many factors including equilibrium
	decreased levels of control success.	female population with regards to
		incomplete fertility.
[42] Esteva and Yang 2005	Models EBS implementation in males	Equation-based population model with
	engineered to have no sperm. Release	density dependent mortality.
	proportion is important.	
[22] Phuc *et al.* 2007	Compared EBS to LBS. They found that EBS at	Time-delayed difference equation model
	low release ratios can increase equilibrium size	with a density-dependent mortality in the
	of adult population, but LBS can result in	aquatic life-stage and based on [43]. The
	eradication. At high release ratio EBS works but	difference between EBS and LBS was
	LBS works better.	characterized in population suppression.
[44] Kean *et al.* 2008	Frequent small releases of EBS moths may be	Discrete-time population model with
	more effective than less frequent releases. They	overlapping generations. This model takes
	also compared how competitiveness of	into account an over flooding parameter
	irradiated males effected control. Models doses	and incomplete sterility.
	of radiation which result in reduced, but not	
	complete sterilisation of males to the benefit of	
	increased mating competitiveness.	
[45] Yakob *et al.* 2009	Modelled LBS, EBS, EFK, and LFK of a	Time-delayed difference equation model
	hypothetical insect population at various	representing the mosquito’s lifecycle with
	release proportions, migrations, density	adult and larval mortality terms.
	dependancies, and fecundities. Found bisex	
	lethal could be preferred over female killing	
	under certain scenarios.	
[46] White *et al.* 2010	Models *Ae. aegypti*, EBS and LBS releases. Found	Population dynamics are modelled with
	control is more effective with fewer males	a time-delayed difference equation model
	released more often than many males released	extended from [43]. EBS and LBS are
	less frequently.	modelled and the dynamics of injected pulses of mosquitoes are reported.
[47] Deredec *et al.* 2011	Models an *An. gambiae* EFK implementation	This work extends a population model
	where the X chromosome in sperm is targeted	by adding HEG dynamics and focuses on
	(and two other transgenic techniques that are	reducing the intrinsic reproductive rate of
	outside the scope of this paper) by release	the female population. Density dependent
	of mosquitoes carrying homing endonuclease	mortality is considered for larvae.
	genes (HEG). Determined the number of	
	individual HEGs targeting essential mosquito	
	genes required at various mosquito	
	reproductive numbers with various homing	
	rates to eliminate a mosquito population.	
[37] Thailayil *et al.* 2011	Models release size of spermless *An. gambiae*	Differential equation model with no explicit
	(EBS) males required at differing rates of	time latency between generations. The
	occurrences where females mate more than	adult female population separated into
	once. Very low levels of remating events were	females who have not mated; mated and
	found to have significant negative effects on	fertile; mated; and infertile. Population
	the ability to control the mosquito population.	persistence was described in terms of the model coefficients.
[48] Dumont and Tchuenche 2011	Found it more effective to have small and	Extensive system of equations which
	frequent releases of EBS males over large	captures population and compartmental
	infrequent releases. Also EBS works better	dynamics.
	when carried out with a larval habitat control	
	program (mechanical control).	
[49] Lee *et al.* 2013	Modelled EBS & LBS in *Ae. aegypti* mosquitoes	Difference equation model similar to [22]
	under endemic and emerging outbreak	but look at an endemic case and emerging
	scenarios. Evaluated various release and	outbreak of mosquito populations.
	intervention-region sizes. Found EBS was	
	always more effective than EBS, though the the	
	magnitude varied by situation.	

## Methods

Four hypothetical SIT implementations were evaluated with an agent-based model for *An. gambiae*. Virtual mosquito agents traversed states that characterized many behavioural and life-stage aspects of the mosquito. Development was determined by a virtual mosquito’s own attributes, namely sex and genotype. All four SIT implementations were represented as a cell-lethal gene expressed in the virtual agents. For EBS, these dynamics can be equivalent to methods that render males with non-viable sperm. Agent-based modelling may be a preferred approach to simulate SIT implementations because it allows us to characterize individual mosquitoes, encode the effect of various genotypes, and model the effects of overlapping generations.

**Figure 1:**
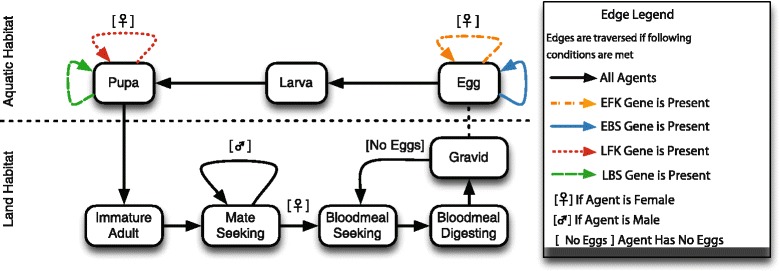
**Virtual mosquito agents transition through a series of states representing life-stage and behaviour aspects of the mosquito’s lifecycle.** Agents carry out these behaviours until death (simulated random mortality, which can happen at any state). The presence of cell-lethal genes halt agent development (indicated by coloured lines). Abbreviations are: EBS, early acting bisex; EFK, early acting female-killing; LBS, late acting bisex; LFK, late acting female killing.

The mosquito agent’s states are represented in Figure 1 while Table 2 outlines the transition rules. Mosquito agents entered the model as eggs and emerged as adults after transitioning through the larval and pupa states. Immature adults developed then sought a mate (females were randomly mated with a mate-seeking male). Males remained mate-seeking while mated females began to complete the gonotrophic cycle. A new agent was created for each egg deposited by an adult female. This agent-based model is fully described in [50] but this section will highlight elements pertaining to the population dynamics where SIT methods can have an important effect.

**Table 2:** **The state transition rules of the model**

**State**	**Duration**	**Exit condition**	**Note**
*Egg*	1 day + *H*_*t*_	None	Reflects incubation and hatch time.
*Larva*	about 12 days	Nighttime	Larval mortality is density dependent and favours
			older larvae.
*Pupa*	1 day + *H*_*t*_	Nighttime	Adult emergence from pupae occurs (6 P.M. to 6 A.M.
			in the simulation).
*Immature Adult*	53 hours	None	
*Mate Seeking*	-	6 P.M. & Female	Mating is 100% successful and mate is assigned randomly.
*Bloodmeal Seeking*	-	Meal Success & Nighttime	Females have a 25% chance of finding a host each hour.
			Bloodmeals take less than 1 hour.
*Bloodmeal Digesting*	36 hours	Nighttime	Agents seek to lay eggs in larval habitats only at night.
*Gravid*	-	Empty Egg Clutch & Nighttime	Agents complete gonotrophic cycles until death.

The model has an hourly time resolution and many agent behaviours are dependent on the time in state and simulated time of day. *H*_t_ is a randomly assigned ‘hatch time’ value described in Equation 1.

**Figure 2:**
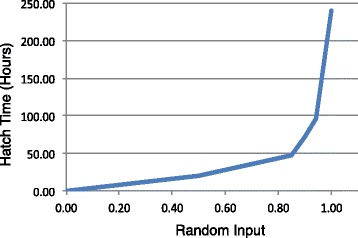
**Visualization of the hatch-time curve given a random input value.**

Egg and pupa state time durations are described as 24 hours plus a ‘hatch-time’ (*H*_*t*_) term. *H*_*t*_ was designed to reflect observations that most eggs hatch within three days, however, some take as long as five days [51]. Hourly *H*_*t*_ determines the amount of time an agent takes to hatch and is designed as a piecewise function of *x*, a variable sampled from a uniform distribution, *U*(0,1), and is defined as:

(1) $$ H_{t}(x) = \begin{cases} 40 \cdot x & : x \le 0.5 \\ 68.57 \cdot x - 10.28 & : 0.5 < x \le 0.85 \\ 480 \cdot x - 360 & : 0.85 < x \le 0.9 \\ 600 \cdot x - 468 & : 0.9 < x \le 0.94 \\ 2400 \cdot x - 2160 & : 0.94 < x \le 1.00 \\ \end{cases}  $$

and seen in Figure 2.

Mortality was experienced differently at each life stage: eggs and pupae were subject to a fixed daily mortality rate of 0.1; adult mortality rate (*M*_*adult*_) was age dependent described as

(2) $$  M_{adult}(n) = { {0.1 \cdot e^{n/25}} \over {1+0.25(e^{n/25}-1)}.}  $$

deriving from [52] where *n* is the age in days. The larva mortality rate (*M*_*larva*_) is density dependent favouring older agents

(3) $$ M_{larva}(n) = 0.1 \cdot e^{{L_{mass}} \over {n \cdot C}}  $$

where *C*, *L*_*mass*_, and *n* are the larval habitat’s carrying capacity, larval biomass and larva age in days, respectively. The larval biomass, *L*_*mass*_, is an age-weighted sum of the larva population:

(4) $$ L_{mass} = \sum\limits_{n=1}^{10}n \cdot L_{n}  $$

*L*_*n*_ is the number of *n* day-old larvae, these equations characterize larval growth as linear with daily age. Larval mortality is largely controlled by the *L*_*mass*_/*C* term in Equation 3. Assuming a constant *C*, larval mortality increases as larval biomass in the habitat (*L*_*mass*_) increases. A larval habitat with fewer larvae will allow for a higher survival rate. This is analogous with previous models [43] but also favours older larvae.

A mosquito agent’s sex and genotype were assigned when it is created. Sex was randomly assigned with an equal probability of male and female. Each agent has two alleles of each gene where one was randomly contributed from the mother and the other was from the father. Cell-lethal genes were dominant and manifested if present. EFK carrying females stopped development in the egg state and EFK carrying males developed as normal. EBS and LBS expressing agents ceased developing in the egg and pupa states respectively.

Mate-seeking females were assigned a mate at random from a distribution of all mate-seeking males weighted by each male’s respective competitiveness values. Competitiveness represents an agent’s ability to mate relative to a true wild-type (if this value is 50%, it is half as competitive as wild-types). Every agent had a mating competitiveness attribute determined by averaging the values of its mother and father. Female competitiveness did not affect mating probability but was considered when determining the female mosquito’s progenies’ values.

### Simulated SIT Campaigns

The simulations captured one year of intervention with an hourly time resolution. A steady agent population persisted for six months prior to the intervention period and an average male population was obtained through 30 consecutive daily observations. Next, homozygous, mate-seeking males were released into the simulation every day for one year. Released males’ mating competitiveness was defined with a simulation parameter. Releases occurred at 3 P.M. and the release number was a fixed amount obtained by multiplying a release proportion parameter and the average wild-type male population before the releases started (this was rounded to the nearest whole number). Note the release number remained the same even if the adult male population was being suppressed.

The modelling approach used in this paper allowed us to record adult and larval population numbers and values within the simulation that contribute to the population dynamics. These numbers were normalized and populations were stratified by sex and genotype. The simulation recorded a fecundity potential measure which relates to the probability that a wild-type female adult will have wild-type female offspring. Given a set of male mosquitoes (**x**_*i*_∈*X*) each has a mating competitiveness (*m*_*i*_), and is wild-type, heterozygous or homozygous (**x**∈{[ 1 0 0],[ 0 1 0],[ 0 0 1]}). Fecundity potential (*F*) is defined as

(5) $$  F = 1 - {\sum{m_{i} * [~0~~0~~1~] \cdot \textbf{x}_{i}} \over {\sum{m_{i}}}} - {{\sum{m_{i} * [~0~~1~~0~] \cdot \textbf{x}_{i}}} \over {2 \cdot \sum{m_{i}}}}.  $$

The first and second numerators return the total mating competitiveness of homozygous and heterozygous males respectively. The coefficient in the second denominator accounts for the fact that half of the female progeny from a wild-type animal mating with a heterozygous animal will carry the cell-lethal gene.

Finally, the simulation reported the mortality term *L*_*mass*_/*C* which is proportional to the density dependent mortality experienced by larvae in Equation 3. Measures were normalized by dividing their values with the output given no SIT campaign (population values were normalized using wild-type numbers).

## Results

SIT campaigns were simulated varying the cell-lethal gene implementation, number of released males, and the mating competitiveness of released mosquitoes. A campaign’s effectiveness was measured in terms of wild-type female adult population suppression as this term relates to the transmission coefficient of vector-borne illnesses. Recall the simulation reached a steady population for 6 months and an average male population was obtained from 30 consecutive daily observations. Then the daily releases started and the number of released males was the release proportion multiplied with the average male population. All reported results are an average of 30 simulations since the models in this paper are stochastic.

**Figure 3:**
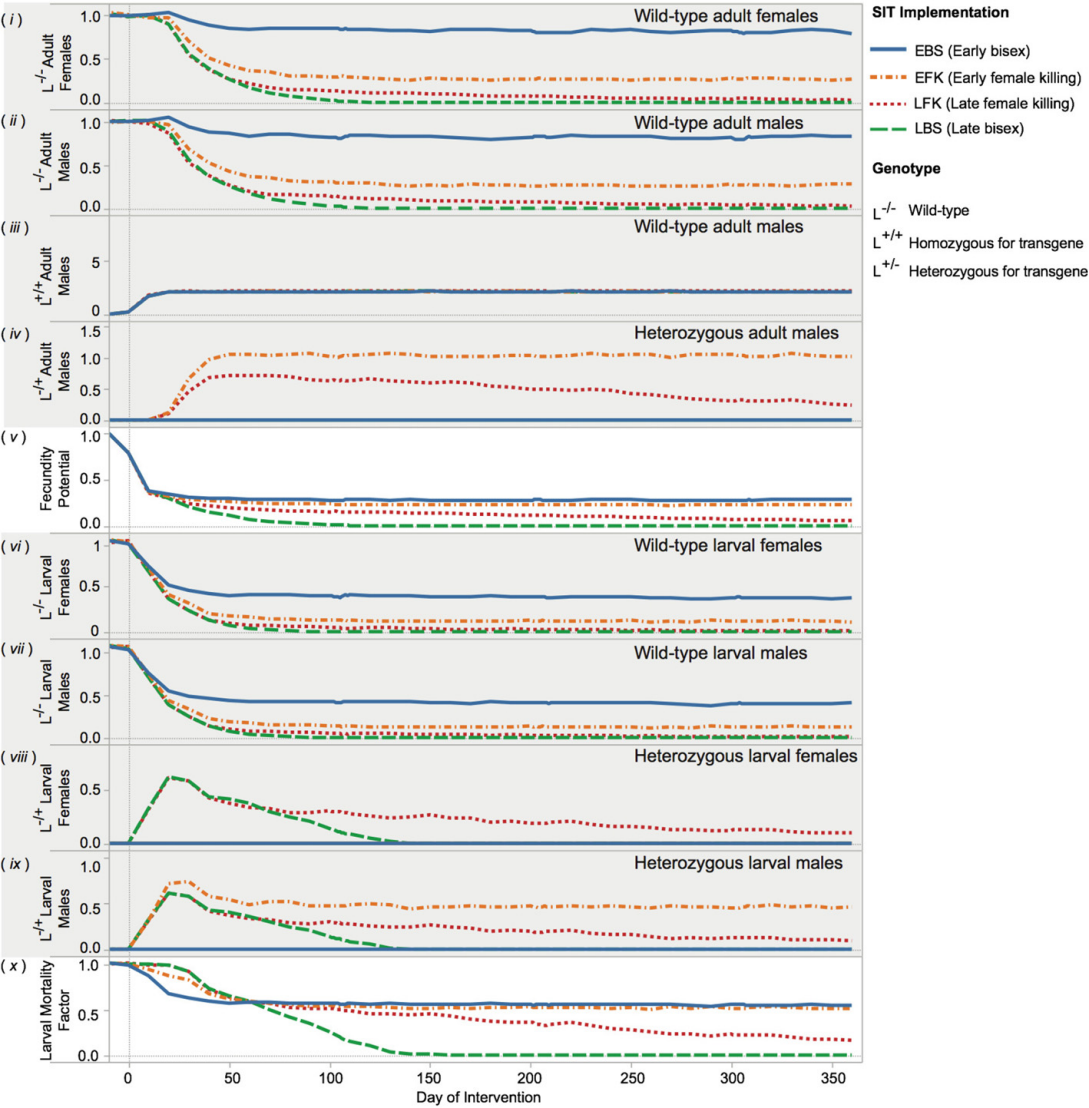
**Simulated daily, fixed-number releases of sterile males with SIT genes that halted growth in all eggs (EBS), only female eggs (EFK), all pupae (LBS), or female-only pupae (LFK).** These plots report the population’s response to the introduction of sterile males at the release proportion of 0.3 from the first release of the campaign (day 0). Released males are as competitive as wild-types in these graphs. From top to bottom, these measures are the number of wild-type adult females (*i*) and males (*ii*); number of homozygous and heterozygous adults males (*iii* and *iv*); the fecundity potential (*v*); wild-type female and male larvae (*vi* and *vii*); heterozygous female and male larvae (*viii* and *ix*); and the density-dependent larval mortality factor (*x*). *L* represents the cell-lethal transgene. Please note that mosquitoes are counted before releases but the fecundity potential measure is calculated after.

**Figure 4:**
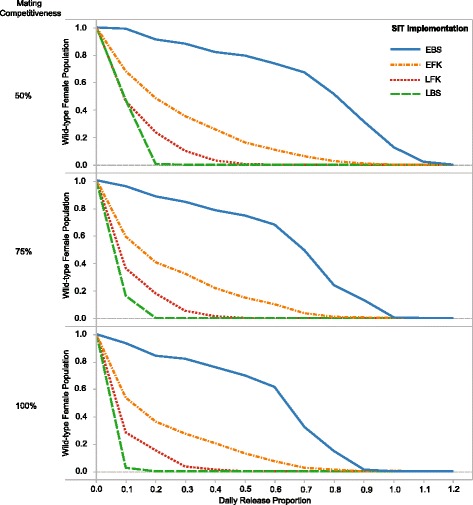
**Population suppression factor reported in terms of released mating competitiveness and daily release proportion for the SIT implementations in the 12th month of the campaign.** A suppression factor of 0.5 means the wild-type population was halved relative to a population with no intervention employed. Competitiveness represents a male agent’s ability to mate relative to a true wild-type male (if this value is 50%, it is half as competitive as wild-types).

**Figure 5:**
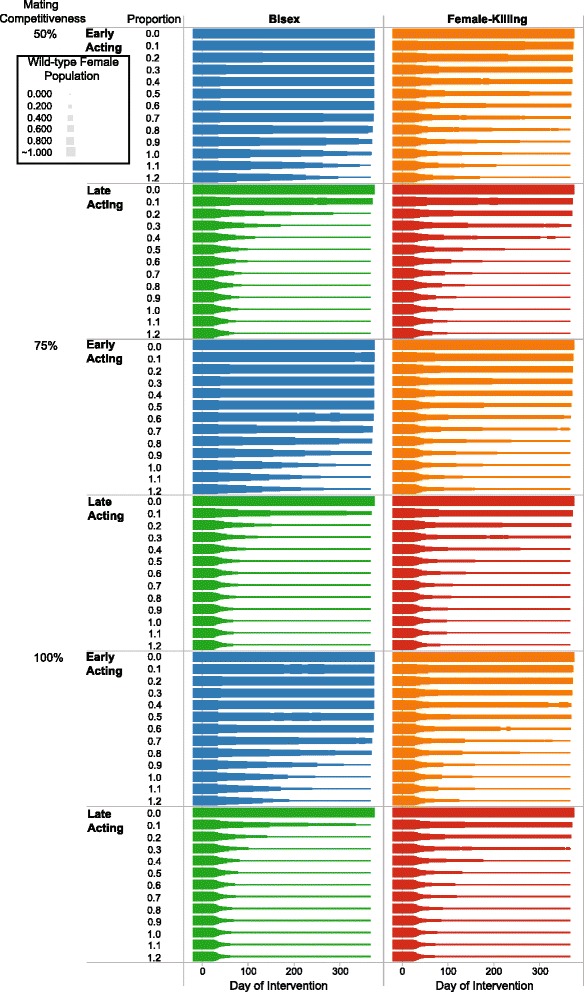
**Population suppression over time for each daily release proportion and cell-lethal gene.** Each cell corresponds to a gene and mating competitiveness and each line to a release proportion. The lines move forward in simulation time and the thickness corresponds to the adult female population. Competitiveness represents a male agent’s ability to mate relative to a true wild-type male (if this value is 50%, it is half as competitive as wild-types).

Plots show measures in terms of release proportion and time after normalising them with average measures given no interventions. Figure 3 is time-series plots showing the simulation behaviour at all time-steps for all populations but with one release proportion. Proportion-series plots show an average measure over 30 consecutive simulated days (Figure 4). Figure 5 demonstrates population suppressions over time for each gene with all release proportions. There are ten measures indexed with Roman numerals on Figure 3 where *L* represents a cell-lethal transgene. Measures are:

**i)***L*^(−/−)^*Adult Females*: population of wild-type adult females.**ii)***L*^(−/−)^*Adult Males*: population of wild-type adult males.**iii)***L*^(+/+)^*Adult Males*: population of released males.**iv)***L*^(−/+)^*Adult Males*: population of males heterozygous for the cell-lethal gene.**v)***Fecundity Potential*: value related to the chance that a female will have wild-type female offspring.**vi)***L*^(−/−)^*Larval Females*: population of wild-type larval females.**vii)***L*^(−/−)^*Larval Males*: population of wild-type larval males.**viii)***L*^(−/+)^*Larval Females*: population of larval females heterozygous for the cell-lethal gene.**ix)***L*^(−/+)^*Larval Males*: population of larval males heterozygous for the cell-lethal gene.**x)***Larval Mortality Factor*: value proportional to the density-dependent larval mortality.

The population dynamics are discussed in terms of initial and final effects. Initial effects detail the population’s response to releases of males with the cell-lethal gene. Final effects are measures in the 12th month of a simulated year-long campaign. Final effects are determined to be important so the discussion is tailored thusly.

### Initial effects

First, the mosquito population’s initial response to the release of lab-reared males in a simulated SIT campaign is considered. Figure 3 shows an example of the response to an SIT release with the release proportion of 0.3. The initial effects, unless otherwise noted, were largely independent of the release proportion.

Several responses were expected given the biological mechanisms that the model captures. Wild-type adult population suppression would not be seen until females mated with a released male, acquired a bloodmeal, developed a clutch of eggs, and her progeny reached adulthood (about 17 days). At this time, heterozygous adult males should first appear for FK methods and the effect on larval mortality would not be observed until the progeny of wild-type mosquitoes mating with released mosquitoes are expected to be larvae (about 5 days) and heterozygous larvae should appear.

These expectations are clearly shown in the charts in Figure 3. Plots *i*, *iii*, and *iv* show the delay from the first release to evident population suppression and the appearance of heterozygous males. The aquatic population’s responses are seen in *vi*, *vii*, *viii*, *ix*, and *x* and demonstrate the delayed effect of releases on larval mortality.

The injection of a cell-lethal gene can disrupt the wild-type population’s equilibrium and this is especially seen in EBS methods where there is a brief, minor increase in wild-type populations (this is seen around day 20 in Figure 3). This is likely due to less density dependent mortality in the larval habitats allowing a few more agents to emerge but this effect is immediately followed by population suppression. Other models have shown that cell-lethal genes can cause an increase in the wild-type population [22]; in the results, this effect is very short.

### Final effects

Whereas the initial effects are largely independent of release proportion, a high release proportion is crucial to population elimination one year following SIT release (Figure 3). Late-acting genes are far more effective than their early-acting counterparts because of their ability to maintain higher larval mortality. Finally, bisex cell-lethal genes are more effective with late-acting implementations.

#### Release proportion

A campaign’s effectiveness is largely dependent on the number of consistently released males on a daily basis. Figure 4 demonstrates that greater population suppression occurs if more sterile males are released. However, there appears to be a point where releasing more sterile males does not cause a noticeable benefit.

Figure 5 shows there is little benefit to releasing LFK males at a higher proportion than 0.7. It is likely that a point of diminishing returns exists for this cell-lethal gene. Though these points are not evident in all the lines of the figure, it is reasonable to assume they exist for all transgenic implementations (assuming a high enough release proportion). Operating above this point could be a waste or misappropriation of resources.

#### Early-acting versus late-acting

It may be reasonable to hypothesize that late-acting SIT implementations are efficient at suppressing the population because they exploit density dependent mortality in the larval habitat. This principle is observed in the results as well. Late-acting genes achieved elimination with fewer released males when compared with their corresponding early-acting counterparts. Early in the campaign, late-acting genes caused the larval mortality to remain higher and this is seen in Figure 3 Plot *x*. This caused more wild-type population suppression and an eventual decrease in larval mortality.

#### Mating competitiveness

The effectiveness of an SIT campaign can be reduced if released males are less competitive when mating. The suppression factor is reported for the last 30 days of a 12 month SIT campaign in terms of daily release proportion and mating competitiveness in Figure 4. In these plots, it is clear that LBS still performs better than LFK because eradication is achieved with the lowest proportion of released males.

The effects of mating competitiveness are less apparent on female-killing methods. This is likely because second generation heterozygous males serve as an additional suppression source and these males are more competitive than homozygous mosquitoes.

#### Female-killing versus bisex lethality

**Figure 6:**
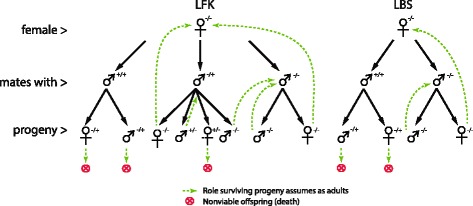
**Possible explanation of why late female killing (LFK) methods are not as efficient in suppressing the wild-type population as late bisex killing (LBS) methods.** In LFK methods, there are two avenues by which a wild-type mosquito can be generated (either through a wild-type mate or, with 50% probability, through a heterozygous mate). The wild-type generation through heterozygous mates can cause the wild population to persist in the environment. With EBS methods, wild-type mosquitoes are only generated with wild-type mates.

The late-acting, bisex, cell-lethal implementation achieved eradication with the fewest number of released males. Late-acting, female-killing genes are often thought to be very effective because they generate a population of males which serve as an additional reservoir for the cell-lethal gene. These results indicate that this population serves as a reservoir for the wild-type gene (illustrated in Figure 6) and reduces a campaign’s effectiveness. This point can be observed in Figure 3 by comparing the fecundity potential (*v*) in relation to heterozygous males (*iv*) and the persistence of the wild-type female population (*i*).

For the early-acting methods, female-killing genes outperformed bisex cell-lethal implementations. EFK allowed for the presence of sterile male larvae and these agents contributed to the density dependent mortality in the larval habitat. That effect was primary in population suppression and was not overcome by the heterozygous male population acting as a reservoir for the cell-lethal gene.

## Discussion

This paper used agent-based modelling to investigate the effect of four SIT implementations on a simulated *An. gambiae* population. The simulations represented daily, fixed-number releases of sterile males with SIT cell-lethal tannsgenes that halted growth in all eggs (EBS), only female eggs (EFK), all pupae (LBS), or female-only pupae (LFK). In the female-killing methods (EFK, LFK), a heterozygous male population survived and could pass a cell-lethal gene to future generations. The lab-reared mating competitiveness was varied along with the male release proportion. The cell-lethal genes were assumed to be 100% effective at only one locus. The conclusions are as follows:

- Population suppression is dependent on the number of released males in a campaign for any cell-lethal gene to a point. After a certain release proportion, the additional males appear to have diminishing returns on population suppression likely due to saturation of the cell-lethal gene. This result is congruent with previous results [44,46,48] but this work shows the dynamic is present with all four cell-lethal implementations tested and using an agent-based modelling technique.
- Late-acting cell-lethality is highly preferred over early-acting because of its ability to exploit density-dependent mortality in the aquatic habitat. A large portion of population suppression occurs within 50 days of the campaign’s beginning. Late-acting genes maintained a higher larval mortality leading to greater population suppression by the end of a simulated year-long campaign. These results are also congruent with previous modelling literature [22,53] but they are demonstrated in an *Anopheline* simulation.
- Late-acting bisex genes are preferred because these genes lead to mosquito elimination with the fewest number of released males. This is a dynamic in SIT systems and may be counterintuitive though presented in [38]. Female-killing methods are thought to be more efficient because the heterozygous male population is an additional reservoir for the cell-lethal gene. However, the converse is that this population can also serve as a reservoir for wild-types and cause a population to persist (this is illustrated in Figure 6).

The findings in this manuscript are not without limitations. For example, using current technologies, 100% lethality is not always ensured, but this is not modelled; instead assuming that 100% lethality will be favoured over sub-optimal mating competition. Further, lethal genes could be introduced at multiple loci, which could increase efficiency considerably. Additionally, *only* males are assumed to be released and no females are released due to sorting difficulties. Clearly, successful female killing strategies would prevent such accidental release of females. Finally, seasonality is not modelled, assuming constant temperatures and larval habitat carrying capacity.

This paper used an agent-based model to simulate four transgenic sterile insect technique implementations and provide feedback on their efficiency in suppressing a mosquito population. Three conclusions were reached from the system’s emergent behaviour; two were in agreement with the previously published literature and one is a novel insight which has not yet seen reported. These results demonstrate the potential of agent-based modelling to validate with other results but also expose novel, emergent behaviour in a complex system.
